# 

**Published:** 1916-07

**Authors:** 


### Publishers' Announcement

A standard bearer is always in front and has men behind with ammunition to back him up—Preparedness. Where the standard is the American flag and the men behind are the people of the United States, the combination is a particularly formidable one. It appropriately appeals to every American citizen to see the American flag properly borne at the front—in the vanguard.

Good, timely and conservative dental journalism is the standard which the "Dental Register" bears and is the standard which it has carried for seventy years. If the standard was not of the kind that lasted, endured, it could not have borne it so long. Nor were it not for the sturdy texture of which it is woven it could not have withstood the fraying tendencies of the breezes.

Like the American flag, the "Dental Register" has added several stars to its field since it began to unfold to the world, and its influence has correspondingly increased. Wherever dentistry is practiced the "Dental Register" is found and has helped in the development of high ideals of practice and skillful administration of the knowledge of dentistry. The "Dental Register" unfolds its banner to the dental profession in every country on the face of the globe. It may appropriately be said that the American flag and the "Dental Register" go hand in hand and shoulder to shoulder. Uncle Sam reads the "Dental Register" and it is on Uncle Sam's library table, and we are proud of it.

### ANNOUNCEMENTS

### CLINICS OF THE NATIONAL DENTAL ASSOCIATION.

The Clinic Committee of the National Dental Association has arranged for two half-day clinics, an Illustrated Lecture Clinic and a Progressive Clinic; and surgical clinics under conductive and general anesthesia for the annual meeting to be held in Louisville, Kentucky, July 25 to 28, 1916.

Illustrated Lecture Clinic, Wednesday, July 26, 1:30 p. m.—These clinics will be presented to the entire membership in attendance at Keith's Theatre, having a seating capacity of over 3,000 people. Time to be occupied by each clinician will vary from twenty to twenty-five minutes. Clinicians and subjects they will present are as follows: Dr. T. P. Hinman—Therapeutic treatment of pulp diseases when the vitality of the pulp can not be retained, including the aseptic management of root canal treatment having special reference to sterile condition of outside of tooth, rubber dam and holder, broaches and cotton employed in the application of remedies, not including the opening up or gaining access to constricted canals. Dr. J. R. Callahan —The technic of root canal filling with rosin and the opening of small and constricted canals. Dr. R. Ottolengui—The technic of root canal filling with gutta percha preparations. Dr. J. D. Patterson—Indications and contraindications for treating teeth involved with pyorrhea alveolaris including statements having reference to the various suggested remedies, including emetin; discussion not to include the technic of instrumentation or the care of the tooth surfaces by the dentist or patient. Dr. W. A. Price—Oral infections with special reference to bacteriology. Dr. E. T. Tinker—Bridge abutments for vital teeth, with exhibition of radiographs showing condition of periapical tissues after the lapse of time. Dr. C. N. Johnson—Practice management, business investments and a dentist's duty to his profession.

Progressive Clinic, Friday, July 28, 9:15 a. m.—These clinics will be held in the balcony of the Armory, which is to be divided into twenty-four rooms, with seats arranged in amphitheatre form. The demonstrations will be given by members of the National Association residing in Michigan, Indiana, Kentucky and Tennessee. The thought back of the plan of confining the progressive clinicians to members of the National District in which Louisville is located is for the purpose of showing the profession at large the type of dentistry being accomplished in this particular section of the country. The subjects to be presented are as follows: Cement Clinic—All forms, the technic of mixing and insertion. Porcelain Clinic—Porcelain jacket crown, its indication and technic of construction. Radiography Clinic—Interpretation of dental radiographs. Local and Conductive Anesthesia Clinic—The technic of making novocain suprarenin solutions, the care of the syringe, needles, etc. The technic of injections of novocain suprarenin for local and conductive anesthesia. Removable Bridgework Clinic—Partial dentures and removable bridgework with description of the various forms of attachment, including the type indicated to engage vital teeth. Impression Taking in Modeling Compound Clinic—Taking impressions in modeling compound with the mouth closed and under normal biting stress. Full Denture Clinic—Full dentures, not including impression taking. Oral Surgery Clinic, Thursday, July 27, 10 a. m. to 12:30 a. m.—Operations under conductive and general anesthesia will be held in the City Hospital. Types of operations to be performed are as follows: Local and Conductive Anesthesia—Cavity preparation, pulp removal and extraction of teeth under novocain suprarenin anesthesia. Antrum operations and alcohol injections for trigeminal neuralgia under novocain suprarenin anesthesia. Removal of impacted inferior third molars under novocain suprarenin anesthesia. Root end amputation of teeth under novocain suprarenin anesthesia. General Anesthesia—Cleft palate, harelip, and such other cases as present. Committee: C. N. Hughes, H. B. Tileston, Wm. H. G. Logan, Chairman, 29 E. Madison Street, Chicago, Ill.

### General Plan for the Twentieth Annual Session of the National Dental Association.

While no sensational changes are contemplated in the general plan of this annual session of the National, to be held in Louisville, July 25, 26, 27 and 28, there are a number of decided improvements which will be inaugurated.

### *Outline and Location of Sessions.*

The first day will begin with registering all members and guests, at the spacious First Regiment Armory rotunda. Such facilities will be provided as will make registration a pleasure. The ladies will register on the mezzanine floor of the Seelbach hotel, which will be their headquarters and from which all entertainments will start.

Opening session will be held in Keith's theater, seating over 3,000 very comfortably. This theater is artificially cooled to the temperature of sixty-five degrees during the summer months, and of its acoustic properties Dr. Thomas Hinman had this to say, after a recent inspection: "Nowhere, with the exception of the Mormon Temple at Salt Lake City, have I observed such wonderful acoustic properties."

All general sessions and some special sectional meetings will be held in this theater, which is diagonally across the street from registration headquarters, one block from Seelbach hotel and one-half block from the Watterson hotel and Hermitage hotel.

The splendid auditoriums of the Seelbach and Watterson hotels will be used for sectional meetings, as well as McCauley's theater, across the street from the Seelbach. Altogether there will be four sectional meetings in progress at the same time, so every phase of modern dentistry will be presented in the most approved manner.

Drs. Hinman, W. H. G. Logan, O. U. King and E. G. Link, of Rochester, while on visits to Louisville, expressed surprise at the fortunate arrangements of buildings in which the sessions could be held, for all hotels and theaters which will be employed are within the radius or length of two (2) city blocks; going from east to west, from Fourth street on Walnut, they are McCauley's theater, Seelbach hotel, Henry Watterson hotel, Keith's theater and First Regiment Armory, where registration, exhibits and progressive clinics will be held.

### *Notable Clinics on Wednesday.*

Perhaps the greatest treat the profession has ever enjoyed will be provided on Wednesday afternoon, when, under the supervision and direction of Dr. W. H. G. Logan, four hours and one-half will be devoted to ''Illustrated Lecture Clinics,'' presented by men of acknowledged authority upon the subjects on which they are to speak.

This is a new feature of a National meeting and affords every member in attendance an equal opportunity to hear and see the same good clinics, and when the profession is assured that Dr. Logan has complete charge of this branch of the meeting, it warrants us to expect something out of the ordinary.

### *More Clinics on Friday Morning.*

The great balcony of the First Regiment Armory will be divided into twenty-seven sections, each to seat from one hundred and twenty-five to one hundred and fifty members and visitors. There will be twenty-seven clinicians, divided into three branches of dentistry, and the outline is so arranged that every man, by remaining in his seat, will see every one of the twenty-seven clinics in the morning allotted for this branch of the work. Much thought and careful study has been devoted to this work and it should be a strong feature in attracting the members of the National to this meeting.

*Another Attractive Feature.*

A feature of special interest to many members of the National will be the joint meeting of the Inter-State Anesthetists, a strong body of both medical and dental men having special interest in anesthesia. A strong program for a joint meeting has been arranged, including the following men: Drs. Long, McMillian, Denman, Brophy, Riethmueller, Thoma and Yager, who will discuss the following subjects: "Oral Operations Under Nitrous Oxide-Oxygen in the Forward Inclined Sitting Posture," "Vapor Anesthesia for Oral Surgery," "Intro-Oral Methods of Local Anesthesia," "Extra-Oral Methods of Local Anesthesia," "Handling Emergencies Under Anesthesia and Analgesia in the Dentists' Office." After this session, which takes place on Wednesday at McCauley's theater, this organization will hold a three days' session in the Red Room of the Seelbach, to which all dentists interested in this useful branch of our healing art are invited.

*Something About Accommodations.*

From some source the expression of fear has reached the local committee on arrangements that Louisville did not have accommodations for a large meeting. To all such fearing friends we submit the following queries:

DO YOU KNOW that Louisville has sixteen splendid hotels, with a total of 3,000 rooms, not to mention the score of lesser hotels for those who prefer to live modestly?

DO YOU KNOW that Louisville's Y. M. C. A., Y. W. C. A., Y. M. H. A. can furnish quarters for hundreds of tourists who prefer that kind of accommodation?

DO YOU KNOW that Louisville has entertained some of the largest conventions in the country in a most acceptable manner, notable among them the International Sunday School Association, with eight thousand registered delegates; the National Canners, with from three to four thousand delegates, have met in Louisville for three consecutive years in preference to other larger competing cities; the great "Saengerfest," which brought thousands to Louisville; Knight Templars, Shriners and a host of the largest gatherings of commercial, political or religious nature in America. This she would be unable to do were it not for her extensive hotel accommodations and genial southern Kentucky brand of hospitality.

*"You'll Feel at Home in Louisville."*

This slogan is ever in the mind of the entertainment committee which is charged with the special responsibility of making every visitor to this convention feel perfectly at home. While this committee is planning to work in harmony with the wishes of the officers of the National, in that no special entertainment will be furnished the male attendants, because of the valuable time it consumes from the regular program, yet they will leave nothing undone to take care of the comforts of all who attend the meeting. Perhaps the greatest service they will render the dentist will be to thoroughly entertain the ladies!

A series of entertainments is being arranged, with the assistance of a ladies' committee, composed of the wife of each local committeeman, which will so completely take up the time of visiting ladies that their husbands will be relieved entirely of the responsibility of providing entertainment. The word comes from this committee, "Tell the boys to bring the ladies and we will take good care of them in true Kentucky style."

Among the entertainments for the ladies are the following: Sight-seeing auto trip with luncheon at the Country Club; boat ride upon the beautiful Ohio river; shopping tour through Louisville's handsome department stores; trip to Fountain Ferry Park, with theater party and lunch, and others.

"You'll feel at home in Louisville."

### ANNOUNCEMENT OF THE RESEARCH INSTITUTE OF THE NATIONAL DENTAL ASSOCIATION.

*Dear Doctor:*—I have just returned from the meeting of the Nebraska State Dental Society and am pleased to report that its members have also unanimously and enthusiastically changed their Constitution, adding $1.00 to the annual dues for the support of the Research Institute of the National Dental Association, which is equivalent to giving a contribution to the Endowment Fund of $9,000, yielding five per cent., since they have 450 members. Besides this, the members of the society present contributed, in cash and pledges, a little over $1,200 to the Building Fund and $600 additional to the current Research Fund. The Nebraska boys are pacemakers in many things pertaining to dentistry.

Since writing you recently, reporting progress, both the current Research Fund and the Building Fund have been steadily growing. Illinois has contributed $500 to each fund, and Mr. H. M. Hanna has given us an additional $1,000 for equipment and running expenses, making the Building Fund at this date over fifty-four per cent. provided, or $27,192. When the other State and Component or District Societies take action, the property will, doubtless, be all or nearly provided for by the time of the National meeting, making it possible to concentrate all effort on the research work and on the raising of the million dollar endowment for its permanent support.

The current Research Fund available from pledges for next year is about $13,000, which, if it could be all realized when due, and collected without expense, would be about two-thirds the amount required to conduct the work on a minimum basis.

There is, apparently, universal endorsement for, and gratification with, the plan of each member of the National Dental Association paying $1.00 dues annually for the support of the research work, which guarantees its perpetual support on a splendid basis, but one which will be constantly enlarged as additional endowments are secured. The urgent, if not imperative, needs of the work require that each state take action at its earliest possible opportunity. This method will supersede and make unnecessary the continual securing of personal pledges, and by this universal distribution the burden becomes a very light one for all. Remember, 18,000 members of the National Dental Association, at $1.00 each per year, is equivalent to $360,000 of the million dollar endowment which we ultimately require and which we confidently expect to get.

Also remember that the property is *your property* and the work is *your work*. Yours very sincerely,

WESTON A. PRICE.

8803 Euclid Ave., Cleveland, Ohio.

